# Effects of vestibular rehabilitation therapy versus virtual reality on balance, dizziness, and gait in patients with subacute stroke: A randomized controlled trial

**DOI:** 10.1097/MD.0000000000033203

**Published:** 2023-06-16

**Authors:** Vishal Sana, Misbah Ghous, Muhammad Kashif, Abdulaziz Albalwi, Rashida Muneer, Mahnoor Zia

**Affiliations:** a Riphah College of Rehabilitation and Allied Health Sciences, Riphah International University, Islamabad, Pakistan; b Department of Physical Therapy, Faculty of Applied Medical Sciences, University of Tabuk, Tabuk, Saudi Arabia; c Islam College of Physical Therapy, Sialkot, Pakistan; d Grand Asian University Sialkot, Sialkot, Pakistan.

**Keywords:** balance, dizziness, gait, subacute stroke, vestibular rehabilitation, virtual reality

## Abstract

Stroke is a neurological disorder with a vascular cause, such as cerebral infarction or hemorrhage, and causes dizziness, balance, and gait impairments in patients. Vestibular rehabilitation therapy (VRT) involves a variety of exercises that can improve balance, gait, and gaze stability in stroke patients by affecting the vestibular system and improving dynamic balance. By providing a virtual environment, the use of virtual reality (VR) can aid stroke patients in improving their balance and gait.

**Objective:**

This study aimed to evaluate the comparative effects of vestibular rehabilitation with virtual reality on dizziness, balance, and gait in patients with subacute stroke.

**Methods:**

The randomized clinical trial involved 34 subacute stroke patients randomly assigned to 2 groups; 1 received VRT and the other VR treatment. To assess mobility and balance, the Time Up and Go test was used, the Dynamic Gait Index was used to assess the gait, and the Dizziness Handicap Inventory was used to determine the level of dizziness symptoms. Each group received 24 sessions of allocated treatment, 3 sessions every week for 8 weeks. Using SPSS 20, both groups pretest and posttest readings were analyzed and compared.

**Results:**

Between the VR and VRT groups, balance (*P*−.01) and gait (*P*−.01) were significantly improved in the VR group, while dizziness was significantly improved in the VRT group with *P* < .001. On within-group comparison, both groups showed significant improvements in balance, gait, and dizziness with *P* < .001.

**Conclusion:**

Both vestibular rehabilitation therapy and VR improved dizziness, balance, and gait in subacute stroke patients. However, VR was more effective in improving balance and gait among patients with subacute strokes.

## 1. Introduction

Stroke occurs from a vascular cause, which can either be a cerebral infarction or any type of hemorrhage. This leads to focal and acute injury of the central nervous system. Strokes lasting longer than 24 hours can cause a disruption in cerebral function, which leads to the development of a variety of clinical symptoms that can either be focal or global in nature. Strokes can lead to death because of its vascular origin.^[1]^

Typical symptoms or signs of stroke comprise sudden unilateral weakness, loss of vision, numbness, double vision, ataxia, speech abnormalities, and vertigo, which is non-orthostatic in nature.^[2]^ Patients with stroke also have problems maintaining postural control and are unable to maintain balance because they have abnormal body imbalance, asymmetrical posture, and difficulty in weight transfer.^[3]^ Approximately 80% of stroke patients are known to be affected by motor impairment, representing a loss or limitation in muscle strength and coordination. Walking ability and balance are greatly affected by motor impairment in the legs.^[4]^ Age, gender, race and ethnicity, and heredity are the nonmodified risk markers of stroke. Generally, though, stroke is a disease associated with aging, and here, the gender-to-stroke risk relationship relies on age. Females are at greater risk of stroke than males at a young age. However, the risk of occurrence of stroke is higher in males than females at older ages.^[5]^ Stroke is the principal cause of death and dysfunction in developing countries, such as Pakistan. Compared with rural areas, urban areas have a higher incidence of strokes in Pakistan. It is estimated that a ratio of 3:2 of stroke incidents occur in rural areas compared with urban areas in Pakistan. People from high-class societies are more likely to suffer from strokes than those from poor working-class societies because of their sedentary lifestyles and easier modes of activity.^[6]^

There have been many different therapeutic approaches created for improving balance, gait, and dizziness, but virtual reality (VR) training,^[7]^ vestibular rehabilitation,^[8]^ robot-assisted training,^[9]^ whole-body vibration training,^[10]^ and treadmill training^[11]^ have received the most attention in the literature.

The vestibular rehabilitation therapy (VRT) program consists of a variety of exercises designed to aid patients in improving their balance, gait, and somatosensory integration, as well as stabilizing their gaze.^[8]^ In addition to facilitating stroke recovery, it also improves the dynamic balance of stroke survivors through its effect on the vestibular system. Studies show that VRT is useful for those with unilateral vestibular impairments and bilateral vestibular loss and for training balance and gaze stability. VRT has also been reported to reduce the risk of falls in patients with vestibular hypofunction and in older adults.^[12]^

The use of VR in rehabilitation has been suggested as an alternative to conventional VRT because of the recent explosion of VR in this field. In vestibular rehabilitation, VR devices have been tested by clinicians because of their potential to achieve substitution, adaptation, and habituation, as well as positive effects on anxiety reduction and a visualization of visual vertigo. VR rehabilitation systems are computer-based programs that provide direct sensorial and dimensional feedback to the person, enabling them to respond, which results in real-time interactions with the environment and provides enhanced enjoyment during an exercise session.^[13]^ In a study by Mao, Chen, and Le Li (2014), VR games were found to be an effective teaching mechanism for patients with balance dysfunction.^[14]^ With advancements in computer technology and VR programs, patients can choose from a variety of environments as a way to enhance their motor skills. Patients with neurological impairments benefit from physical activities using VR programs. Moreover, VR training plays an important role in improving motor recovery and brain reorganization in stroke patients, which are vital for recovering functional movements.^[15]^ In addition, VR improves balance, reduces the risk of falls, and enhances vertical perception in stroke patients.^[16]^ When compared with VRT in patients with subacute stroke, VR has rarely been studied. Hence, the current study aimed to determine the effectiveness of VRT and VR for patients with subacute strokes. Furthermore, the study demonstrates which intervention improves dizziness, balance, and gait more effectively.

## 2. Methods

### 2.1. Participants and methods

The participants were recruited from Railway General Hospital, Rawalpindi, and Allied Hospital, Faisalabad, using convenience sampling methods. All of the participants were volunteers. Subacute stroke patients with a duration of 1 to 6 months, both male and female subacute stroke patients aged 40 to 70 years, patients with a positive head thrust test, and patients with vestibular disorders (dizziness, vertigo, 1–4 score on Modified Rankin score, Mini-Mental State Examination score > 25) were included; patients who presented with neurological conditions other than stroke and those unable to provide informed consent were excluded. The study was approved by the Research and Ethics Committee of Riphah International University Pakistan (Riphah/RCRAHS/REC/00830), and the trial was registered with ClinicalTrials.gov: NCT04771169. Following an explanation of the research protocol, written informed consent forms were collected from the participants. This study followed the CONSORT 2010 checklist of information to include when reporting a randomized trial (available at http://www.consortstatement.org/media/default/downloads/CONSORT%202010%20Checklist.pdf).

### 2.2. Sample size

Based on a previous study using 95% confidence intervals, 80% power, and a mean value of the time up and go (TUG) outcome measure of 20.50.5 (8.1) for the experimental group and 29.6 (10.5) for the control group, using G* power (version 3.1.9.2) the sample size calculated was 34.^[17]^

### 2.3. Randomization

**Figure 1.**
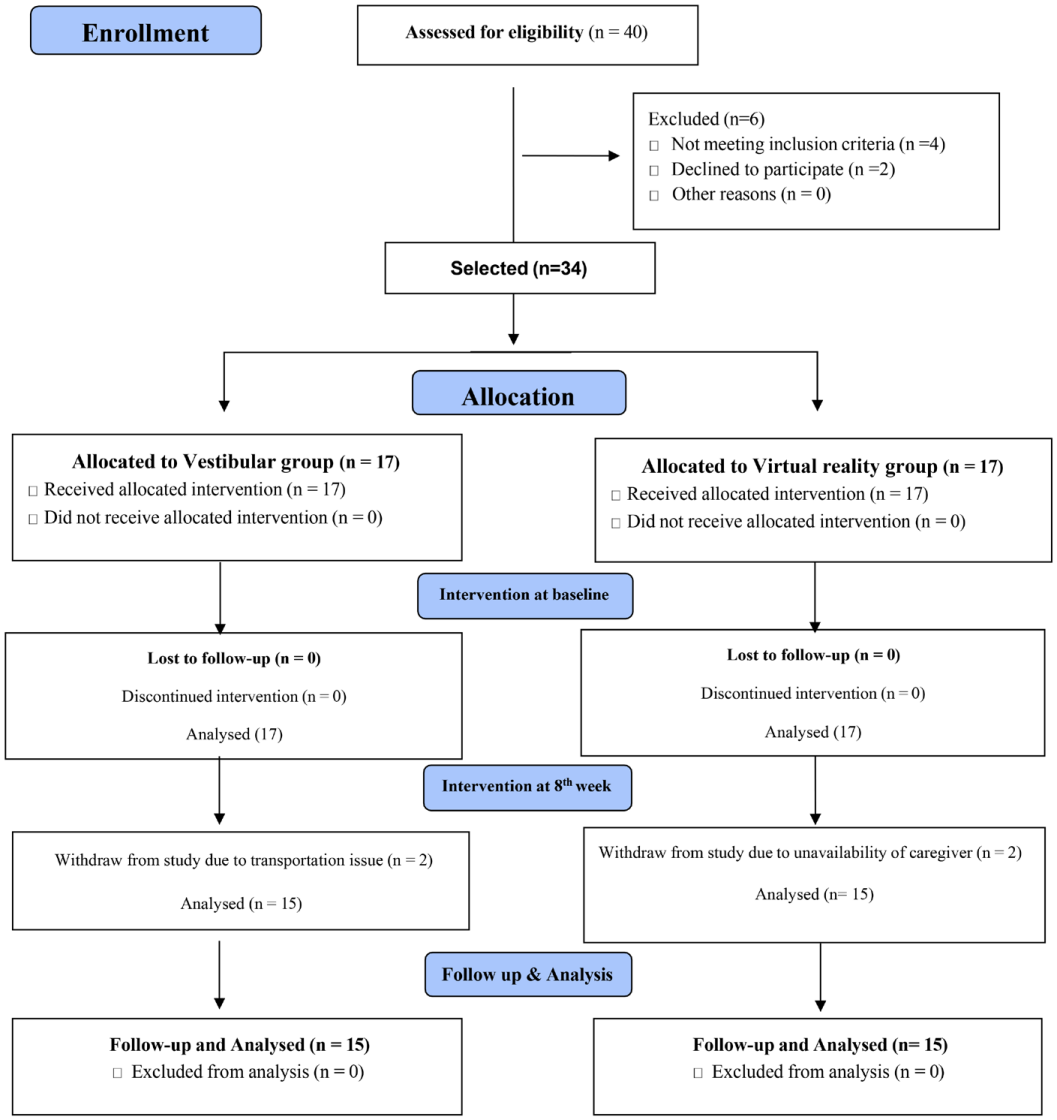
CONSORT study flow diagram.

This single-blinded trial was conducted with 34 patients with subacute stroke, where block randomization with a concealed allocation method was used to assign participants to either the VRT or VR treatment. The primary author (Principal Investigator) allocated a number to the study participants. Data on demographics and clinical characteristics were obtained from the participants and are presented in Table 1 and study flow diagram is presented in Figure 1.

**Table 1:** Characteristics of the participants.

Variables	Group A (vestibular)			Group B (virtual reality)			*P* value
	Frequency (n)	Percentage (%)	Mean ± SD	Frequency (n)	Percentage (%)	Mean ± SD	
Mean age			54.87 ± 9.47			49.73 ± 5.81	.87
Gender							
Male	5	33.3		8	53.3		
Female	10	66.7		7	46.7		
Hemiplegic side							
Right side	8	53.3		6	40		
Left side	7	46.7		9	60		
Duration of stroke							
1–3 mo	9	60		5	33.3		
4–6 mo	6	40		10	66.7		
Comorbidities							
Diabetes mellitus	4	26.7		4	26.7		
Cardiac problems	7	46.7		5	33.3		
Others	4	26.7		6	40		
Modified Rankin score (mRS)							
Slight disability	3	20	2.80 ± 0.41	10	66.7	3.43 ± 4.44	.09
Moderate disability	12	80		5	33.3		
Vestibular loss							
Unilateral	9	60	1.26 ± .45	12	80	1.20 ± .41	.408
Bilateral	6	40		3	20		

### 2.4. Outcome measures and assessment

Timed Up and Go Test. The TUG (Timed Up and Go) test is a measure of mobility and balance in older adults. TUG was used to assess mobility and balance in the current study. The score range for the TUG test can vary depending on the population being tested and the criteria used for evaluation, but a general range is 10 to 20 seconds for older adults without mobility issues, and more than 20 seconds for those with mobility issues. It is important to note that this test should be performed and interpreted under the supervision of a healthcare professional. Cut of scores indicating risk of falls by older stroke patients is 14 seconds.^[18]^

Dynamic Gait Index: The dynamic gait index (DGI) measures gait and balance in individuals with mobility impairments. This test assesses balance, stability, and coordination, among other aspects of gait. Tasks are scored from 0 to 4, with 0 denoting difficulty and 4 denoting ease of performance. The sum of the scores from all 8 tasks is the final score, which is 0 to 32. The higher the DGI score, the better the gait and balance performance. DGI was used to assess the gait of the patients with stroke in the current study.^[19]^

Dizziness Handicap Inventory: The dizziness handicap inventory (DHI) is a self-administered questionnaire used to evaluate the impact of dizziness on an individual’s daily life. The DHI consists of 25 questions that assess various aspects of dizziness such as physical, emotional, and functional handicap. The questions are scored on a 5-point Likert scale, with higher scores indicating a greater impact of dizziness on the individual’s daily life. The final score ranges from 0 to 100, with a higher score indicating a greater level of handicap due to dizziness. The DHI was used to evaluate the level of dizziness symptoms among stroke patients enrolled in this study.^[20]^

A trained physiotherapist (assessor) with 10 years of experience in neurological rehabilitation who was blinded to trial conducted the assessment at baseline and at post intervention in the 8th week.

### 2.5. VRT protocol

#### 2.5.1. To improve gaze stability.

Patients were instructed to keep their eyes on a target that was stationary while being asked to continuously move their head horizontally, that is, side to side, for 1 minute. Afterward, they were instructed to perform the head movements vertically, that is, up and down, while the target remained stationary. The patients repeated their head movements as fast as they could while keeping their focus on the target. The patients were continuously monitored to ensure that they maintained gaze stability during each task. The patients were then instructed to keep their heads stationary and follow a slow-moving target. The target kept moving in different directions, including left, right, upward, and downward, while the head was held in a neutral position. The study duration was the same for all participants. Initially, these exercises were performed for 1 to 4 weeks; after that, for 5 to 8 weeks, they were advanced by adding more challenging tasks, such as standing from sitting to standing, separating feet and then combining them, and walking between them.^[21]^

#### 2.5.2. To improve balance.

While performing neck rotations to the right and left and shifting weight in a forward and backward direction and from side to side, the patients were instructed to maintain their balance. Other exercises were with patients standing on a padded mat with their eyes moving from right to left with their feet together. After keeping their feet together and eyes closed and then in the same position, they moved their head from the right side to the left. The patients were asked to sit on a chair or on the edge of a bed, and then, they stood up and sat down while moving their eyes from right to left. The same movement was performed with head movements to the right and left. After that, the patients performed the same task with head movements to the right and left with their eyes closed. The above-mentioned exercises were then performed while standing on a padded mat.^[22]^

#### 2.5.3. To improve gait training.

Gait exercises that were carried out during a period of 1 to 4 weeks included walking at different speeds, walking, and then turning, walking forward and backward, sideways walking, walking in a circle, walking with head movements in the horizontal and vertical positions, walking, and talking. For 5 to 8 weeks, the level of these exercises was advanced by adding more challenging conditions, that is, changing base of support by moving from a firm surface to a foam surface and by going open eyes to closed eyes. A total of 24 sessions with 3 sessions per week for 8 weeks were performed. The patients exercised for 30 minutes during each session. The patients receiving VRT also had general physical therapy treatment for 30 minutes per day, 3 days per week for 8 weeks.

### 2.6. VR treatment protocol

The VR training program consisted of the Nintendo Wii console (RVL001, Nintendo, EUR), Wii Balance Board, Nintendo Wii remote control (RVL-003), and Wii Fit Plus software (Nintendo D-63760 GroBostheim).

**Figure 2.**
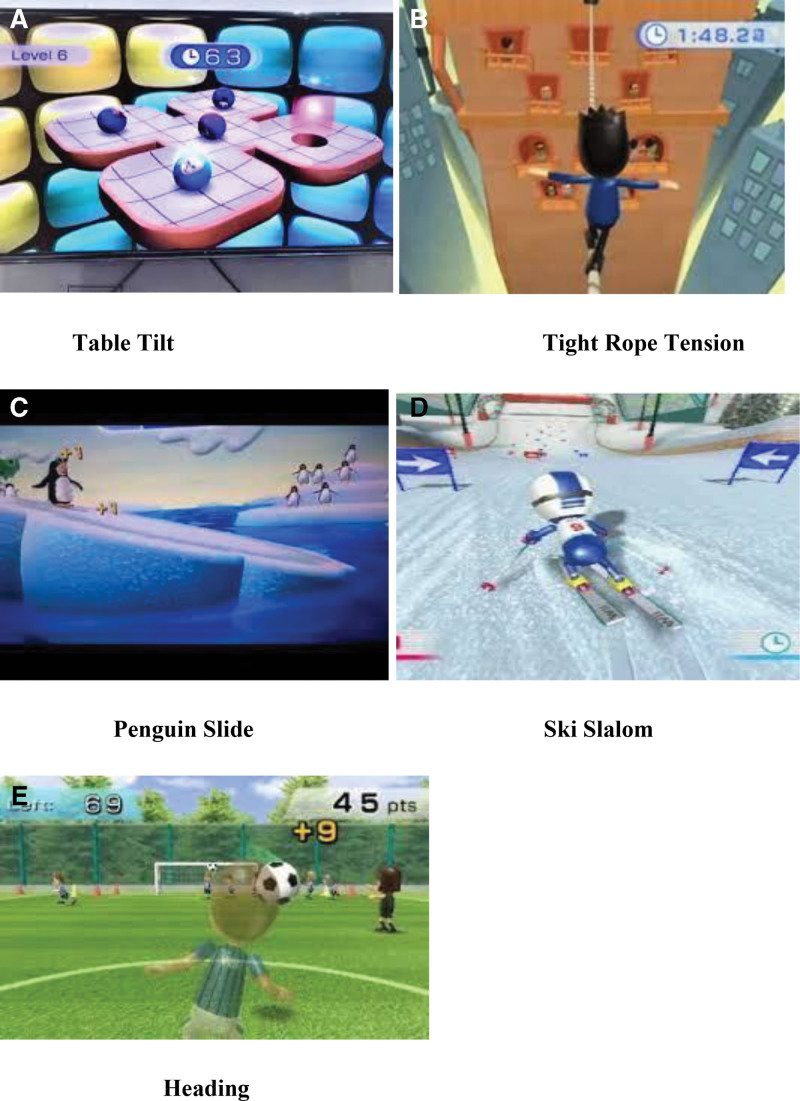
Virtual reality training games.

The patients were instructed to stand on the Wii Balance Board. The Wii Balance Board had a connection to the Wii using Bluetooth. The movements of the patients were shown on the LED screen by an avatar representing the self-expressions of the patient. During the session, the patients stood on the Wii board in front of the LED screen, which was placed at a distance of 2 meters. The patients performed the session under complete supervision to ensure their safety. If any abnormal or jerky movement or muscle spasm was observed before or during the session, the patient was immediately lowered with assistance. When the patient successfully completed the program, the level was advanced. The progression from beginner to advanced level was based on the patient’s individual ability. The games used in the VR session were selected based on the patient’s condition. The levels of complexity were decided according to the progress in the patient’s condition based on suitable levels for dizziness, balance, and gait improvement. The games consisted of Table Tilt, Tightrope Tension, Penguin Slide, Ski Slalom, and Heading.^[15]^ (Fig. 2) A total of 24 sessions with 3 sessions per week for 8 weeks were performed. The patients had VR training for an overall duration of 20 minutes, here with a 5-minute warm-up period before the exercise and 5-minute cooldown period after the exercise.

### 2.7. Adverse events report

If any discomfort or worsening of their condition occurred during the study, the participants were asked to report this to the research team. An adverse event report was made to the participant’s physician in the event of an adverse event. In both groups, the participants were asked not to join in any other exercise regimen, whether formal or at home.

### 2.8. Statistical analysis

Statistical analysis was done using SPSS 20. The Shapiro–Wilk test was used to assess the normality of the data. The data of the TUG, the DHI, and the physical, emotional, and functional domains of the DHI did not follow a normal distribution because the *P* value was <.05. However, the DGI was normally distributed because the *P* value was >.05. For the TUG, the DHI, and the physical, emotional, and functional domains of the DHI, the Mann–Whitney *U* test was used for between-group analysis and the Wilcoxon test for within-group analysis. In terms of the DGI, an independent t-test was used for between-group analysis, and a paired *t* test was used for within-group analysis. Because of loss at follow-up, the VRT group had 15 participants and VR group also had 15 participants.

## 3. Results

A total of 40 participants were recruited, of which 34 met the eligibility criteria. The participants were allocated to the VRT or VR groups. Two participants from the VRT group and 2 from the VR group dropped out because of transportation concerns or caregivers unavailability, leaving 15 in each group. The demographics of the study show that the mean ± standard deviation of the age of the participants of VRT group was 54.87 ± 9.47years and VR group was 49.73 ± 5.81 with no significant difference at baseline with *P* value = .87. Out of the total participants, 13 (43.3%) were male and 17 (56.7%) were female. According to the modified Rankin score, 56.70% had moderate disability and with no significant difference at baseline with *P* value = .09 (Table 1).

**Figure 3.**
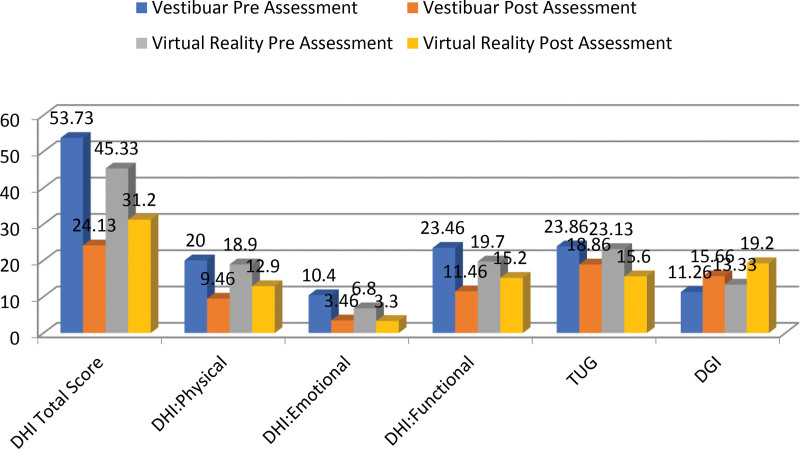
Difference between the vestibular and virtual reality groups on TUG, DHI, DHI: physical, DHI: emotional, DHI: functional, DGI. DGI = dynamic gait index, DHI = dizziness handicap inventory, TUG = time up and go.

At baseline, the TUG scores of both groups showed impaired balance in the stroke patients. When analyzing the between-group scores for the TUG, the participants in the VRT group and VR group showed significant improvement in balance, with *P* value < .01 at the 8th week. Similarly, significant improvement was observed in dizziness for DHI in both the VRT and VR groups, with *P* value < .01 at the 8th week. A significant improvement was observed in the physical, emotional, and functional domains of DHI between both the VRT and VR groups, with *P* value < .01 (Fig. 3).

When analyzing the within-group scores, a significant improvement was seen in the VRT VR groups in terms of the TUG and DHI a with *P* < .001. A significant improvement was observed in both the VRT and VR groups for the physical, emotional, and functional domains of the DHI, with *P* < .001 (Table 2).

**Table 2:** Within-group comparison of the TUG and DHI outcome measures.

Variables	Groups	Assessment	Mean rank	Median IQ	Z-value	Effect size	*P* value
TUG	VRT Group	Pre-value	8	24.0 (3.0)	3.455	0.59	.001
		Post-value	8	19.0 (3.0)			
	VR Group	Pre-value	8	22.0 (6.0)	3.415	0.58	.001
		Post-value	8	15.0 (6.0)			
DHI	VRT Group	Pre-value	8	54.0 (18.0)	3.41	0.58	.001
		Post-value	8	22.0 (16.0)			
	VR Group	Pre-value	8	42.0 (16.0)	3.419	0.58	.001
		Post-value	8	32.0 (8.0)			
DHI: Physical	VRT Group	Pre-value	8	20.0 (2.0)	3.418	0.58	.001
		Post-value	8	8.0 (6.0)			
	VR Group	Pre-value	8	20.0 (4.0)	3.453	0.59	.001
		Post-value	8	12.0 (4.0)			
DHI: Emotional	VRT Group	Pre-value	7.5	12.0 (10.0)	3.305	0.56	.001
		Post-value	7.5	2.0 (2.0)			
	VR Group	Pre-value	7	8.0 (6.0)	3.221	0.55	.001
		Post-value	7	2.0 (2.0)			
DHI: Functional	VRT Group	Pre-value	8	24.0 (8.0)	3.422	0.58	.001
		Post-value	8	12.0 (4.0)			
	VR Group	Pre-value	7.5	15.0 (6.0)	3.359	0.56	.001
		Post-value	7.5	14.0 (4.0)			

DGI = dynamic gait index, DHI = dizziness handicap inventory, TUG = time up and go, VR = virtual reality, VRT = vestibular.

Between groups comparison showed significant improvement in DGI score with a *P* value of .00. For the within-group analysis, for the VR and VRT groups significant improvement in terms of DGI with *P* < .001with a *P* value of .001. (Table 3).

**Table 3:** Within & between-group comparison of DGI outcome.

Within Groups comparison				Between groups comparison	
Groups	Variables	Mean ± SD	*P* value	Mean ± SD	*P* value
VRT Group	Pre-DGI	11.26 ± 2.86	.001	11.26 ± 2.86	.038
	Post-DGI	15.66 ± 2.69		15.66 ± 2.69	
VR group	Pre-DGI	13.3 ± 2.28	.001	13.3 ± 2.28	.001
	Post-DGI	19.2 ± 2.11		19.2 ± 2.11	

DGI = dynamic gait index, SD = standard deviation, SD = standard deviation, VR = virtual reality, VRT = vestibular.

## 4. Discussion

The current study was a randomized clinical trial that compared vestibular rehabilitation with Virtual Reality (VR) on dizziness, balance, and gait in patients who had suffered a subacute stroke. The study found significant improvements between groups for the TUG, DHI, physical, and emotional domains of the DHI and DGI. These outcome measures showed significant improvement within the groups based on within-group analysis.

Gui bin Song (2015) conducted a study to determine the effects of VR on balance, gait, and depression in stroke patients. The study reported a significant improvement in balance in terms of the TUG score (*P* < .05).^[23]^ The finding of this study is consistent with the current study, which has also reported a significant improvement in balance in terms of the TUG (*P* < .05) in the VR group. Karasu et al^[17]^ conducted a study to determine the effectiveness of Wii-based rehabilitation in stroke which recruited 23 participants with a follow-up of 8 weeks using the TUG test. The results show no significant improvements between the groups in terms of TUG.^[17]^ The findings of this study are in contrast to the current study, which has shown significant improvements in balance in terms of the TUG (*P* < .05) in the VR group.

According to Meldrum et al, a study was carried out to compare the effectiveness of VRT using VR rehabilitation in stroke patients over an 8-week period. The study concluded that VR-based rehabilitation showed significant improvement in gait and balance compared with vestibular rehabilitation alone in terms of DGI.^[4]^ The findings of the study are consistent with the current study, which recruited 30 participants and for which the time duration was 8 weeks. The current study also has indicated that VR showed significant improvements in the DGI (*P* < .05) compared with the vestibular group.

In 2019, Viziano et al^[24]^ conducted a study to investigate the long-term effects of VRT and head-mounted gaming task procedures. The effects of VRT were observed in the DGI and DHI. The results of the study show that there were significant improvements in DHI (*P* < .05) and DGI (*P* < .05) in both groups. The results of the study are in line with those of the current study in terms of the physical domain of the DHI (*P* < .05) and DGI (*P* < .05) because there was significant improvement in terms of dizziness and balance of patients.

Mitsutake et al^[21]^ conducted a study to investigate the effects of VRT on gait performance in stroke patients; the study determined the effects of this intervention on stroke patients using the TUG and DGI. The study concluded that there was no significant improvement in the TUG (*P* > .05), with no Significant improvement in balance using vestibular rehabilitation. However, the DGI showed Significant improvement (*P* value < .05), with improvement in the gait of the patient. The findings of the study are consistent with the current study in terms of DGI, which also reported a significant improvement (*P* value < .05) for vestibular rehabilitation. However, in terms of the TUG, the findings contrast those of the present study because the present study reported a significant improvement in balance using vestibular rehabilitation (*P* value < .05). According to Meldrum et al^[25]^ (2015), during the gait cycle, the vestibular system is active at certain points and has a role dependent on the phase of the gait cycle. These roles include double support, changing direction, and termination of steps. Hansson et al found that vestibular rehabilitation reduces dizziness and vertigo in neurological conditions, such as stroke; hence, patients with gait problems following a stroke also benefit from it.^[22]^

This study was the first of its kind to compare the application of VRT with virtual reality in stroke patients. Since the COVID-19 pandemic occurred during this study, patients were hesitant to enroll and follow-up properly. Only subacute stroke patients were included in the study; acute and chronic stroke patients were not included. There were dropouts in the study due to difficulty adhering to the protocol. Moreover, the baseline differences might contribute to between-group differences in the effects of interventions. Further research should be conducted on the long-term effects of vestibular rehabilitation and virtual reality on strokes and other neurological diseases. Clinicians should use vestibular rehabilitation as a treatment protocol for patients with stroke or other neurological conditions in their clinical practice. Moreover, Further research is needed to establish the benefits of VR training and VRT on rehabilitation in people with acute and chronic strokes.

## 5. Conclusion

Based on the findings of the current study, both intervention types appear to be effective and demonstrate significant benefits, but VR has a greater effect on improving balance and gait in people with subacute stroke when compared with VRT, which has a greater impact on reducing dizziness.

## Author contributions

**Conceptualization:** Vishal Sana, Misbah Ghous, Muhammad Kashif.

**Data curation:** Vishal Sana, Muhammad Kashif.

**Formal analysis:** Vishal Sana, Rashida Muneer, Mahnoor Zia.

**Funding acquisition:** Vishal Sana, Rashida Muneer, Mahnoor Zia.

**Investigation:** Vishal Sana, Abdulaziz Albalwi, Rashida Muneer.

**Methodology:** Vishal Sana, Muhammad Kashif, Abdulaziz Albalwi, Mahnoor Zia.

**Project administration:** Misbah Ghous, Muhammad Kashif.

**Resources:** Vishal Sana, Abdulaziz Albalwi, Rashida Muneer, Mahnoor Zia.

**Supervision:** Misbah Ghous, Muhammad Kashif.

**Software:** Abdulaziz Albalwi, Rashida Muneer, Mahnoor Zia.

**Validation:** Vishal Sana, Misbah Ghous, Muhammad Kashif.

**Visualization:** Vishal Sana, Misbah Ghous, Muhammad Kashif.

**Writing – original draft:** Vishal Sana, Misbah Ghous, Muhammad Kashif.

**Writing – review & editing:** Vishal Sana, Misbah Ghous, Muhammad Kashif.
